# Short incision versus minimally invasive surgery with tool-kit for carpal tunnel syndrome release: a prospective randomized control trial to evaluate the anterior wrist pain and time to return to work or activities

**DOI:** 10.1186/s12891-022-05663-5

**Published:** 2022-07-25

**Authors:** Pichitchai Atthakomol, Sitthikorn Kaensuk, Worapaka Manosroi, Apiruk Sangsin, Montana Buntragulpoontawee, Siam Tongprasert

**Affiliations:** 1 Department of Orthopaedics, Faculty of Medicine, Chiang Mai University, Chiang Mai, Thailand; 2 Clinical Epidemiology and Clinical Statistic Center, Faculty of Medicine, Chiang Mai University, Chiang Mai, Thailand; 3 Division of Endocrinology and Metabolism, Department of Internal Medicine, Faculty of Medicine, Chiang Mai University, Chiang Mai, Thailand; 4 Department of Rehabilitation Medicine, Faculty of Medicine, Chiang Mai University, Chiang Mai, Thailand

**Keywords:** Short incision, Minimally invasive surgery, Carpal tunnel syndrome, Randomized controlled trial, Anterior wrist pain, Time to return to work, Time to return to activities of daily living

## Abstract

**Trial design:**

The prospective randomized controlled trial.

**Background:**

This study compares outcomes in terms of early postoperative anterior wrist pain and time to return to work or activities of daily living of patients who underwent carpal tunnel syndrome (CTS) release with short incision and those who had minimally invasive surgery (MIS) with CTS kits.

**Methods:**

A total of 24 patients diagnosed with primary CTS confirmed with electrodiagnosis at an academic university hospital were randomly assigned into one of two groups of 12 patients each: a short incision group and an MIS with tool-kit group using computer-generated block randomization (block of four). Sequentially numbered, opaque, sealed envelopes were used in the allocation concealment process. In the short incision group, skin was incised longitudinally from Kaplan’s line to the area distal to transverse wrist crease (2.5–4.0 cm) while in the tool-kit group, an incision of less than 2.5 cm. was made using special MIS-CTS kits. Primary outcomes evaluated include visual analogue scale (VAS) measurement of pain intensity in the anterior carpal area both while at rest and while conducting daily activities at the 2nd week postoperatively as well as the time to return to activities of daily living and work. Improvement in the Michigan hand questionnaire (MHQ) score, a secondary outcome, was also measured at the 2nd week postoperatively. Patients, allocator and outcome assessor were blinded.

**Results:**

Demographic data, including preoperative electrodiagnostic severity and occupation, were similar in the two groups. There were no significant differences in terms of VAS of the early postoperative anterior carpal area at rest (*p* > 0.99), while conducting daily activities (*p* = 0.89) and time to return to activities of daily living (*p* = 0.46) and work (*p* = 0.24). The MHQ score improvement at the 2nd week postoperatively showed no significant difference between the groups (*p* = 0.95). The MIS wound length in the tool-kit group was significantly shorter than in the short incision group (1.95 vs 2.92 cm, *p* < 0.01).

**Conclusions:**

There is no difference in early postoperative anterior wrist pain, time to return to work or to activities of daily living between the surgical techniques. Short incision is recommended for benefit in term of cost-effectiveness, while MIS with tool-kit could be preferred in patients who concerned in cosmetic appearance between the surgical techniques.

**Trial registration:**

www.clinicaltrials.in.th (TCTR20200530003). Registered 30 May 2020.

**Supplementary Information:**

The online version contains supplementary material available at 10.1186/s12891-022-05663-5.

## Introduction

Carpal tunnel syndrome (CTS) is the most common compressive neuropathy in the upper extremity, causing pain, numbness, tingling and weakness of the hand and leads to long-term work disability [1, 2]. The prevalence of clinical CTS in the general population has been reported to be up to 3.8% [3, 4]. It is more common in middle-aged females and in specific populations including workers whose occupation involves forceful and repetitive wrist motion and employees in industrial settings [5–7].

There is evidence that surgical release provides better results than conservative treatment in terms of reduced symptom severity and improved function at 6 months after treatment [8–10]. Meta-analyses of previous publications have reported no significant difference in terms of symptom relief or functional improvement in the short- or long-term between open and endoscopic carpal tunnel release [11–13]. However, earlier return to work or activities of daily living and lower scar-related complications were found in smaller incision group with large difference of surgical wound length (open versus endoscopic carpal tunnel release or limited-incision versus standard-incision) [11–15].

The standard incision is generally defined as a long, curvilinear palmar incision [16, 17]. Presently, a short incision confined to the palm of the hand requiring no special instruments is commonly used procedure [18]. A minimally invasive surgery (MIS) technique with special tools to improve visualization is used in cases where a smaller incision is required [19]. Unlike the endoscopic technique, these surgical techniques do not require special training and does not involve a long learning curve [20].

A recent meta-analysis reported that a limited incision allows an earlier return to normal activities than the standard incision [15]. There were, however, some methodological flaws in that meta-analysis, e.g., it included retrospective studies and non-randomized studies which contained some bias [17, 21–23]. Additionally, most of the studies in that meta-analysis compared the standard long curvilinear palmar incision proximal to the distal wrist crease (wound lengths up to 7 cm.) to the MIS technique which has a much smaller surgical incision length that leads to decreased anterior wrist pain (scar tenderness, pillar pain) and thus to the ability to conduct activities of daily living and return to work sooner [18, 24–26]. Randomized controlled trials comparing outcomes between short incision and MIS with tool-kit in terms of early postoperative wrist pain and time to return to work and activities of daily living are still lacking. Those studies may provide different results due to the relatively small difference in surgical incision length.

The aim of this study is to compare early postoperative anterior wrist pain and time to return to work or activities of daily living between short incision and MIS with tool-kit in patients who underwent CTS release.

## Material and methods

### Study design and patients

A prospective, single center, randomized, parallel-group superiority trial was conducted at an academic university hospital between June 2020 and December 2021 following the CONSORT (Consolidated Standards of Reporting Trials) statement [27]. A total 24 patients who underwent carpal tunnel release during that period were included in the trial which was approved by the local Institutional Review Board (ORT256206310) and registered in the Thai Clinical Trial Registry (TCTR20200530003, 30/05/2020). Written informed consent was obtained from all patients prior to enrollment and randomization. Carpal tunnel syndrome patients who had been diagnosed by Orthopaedic hand staff according to the criteria of practice parameters for carpal tunnel syndrome [28] confirmed both by diagnosis and by grading severity via electrodiagnosis measurement evaluated by a Rehabilitation Medicine board-qualified staff member and who were scheduled for surgical treatment were included in the trial. All patients aged at least of 18 years who provided informed consent and who were able to communicate orally and to read and write were screened for inclusion. The exclusion criteria included a history of carpal tunnel or wrist surgeries in the affected hand, local anesthetic drug and Non-steroidal anti-inflammatory drugs or opioid allergy, a history of hand or wrist fracture, underlying rheumatoid arthritis, gout, hypothyroidism, active cervical radiculopathy, coagulation disorder, other concomitant nerve entrapments such as Guyon’s canal or cubital tunnel syndrome, concomitant hand or wrist disorders, e.g., stenosing tenosynovitis or osteoarthritis of the finger or hand at the time of enrollment, and pregnancy. All patients were enrolled by Orthopaedic hand staff (PA).

Demographic data, including age, sex, dominant hand, affected hand, graded severity (mild, moderate, severe due to electrodiagnosis measurement) [29], underlying diabetes mellitus, occupation (manual laborer, officer, housewife, unemployed, retired) were collected. Intention to treat analysis which interpreted the result according to the group they were originally assigned was performed.

### Randomization and trial intervention

Patients were randomly assigned to receive one of the two interventions, either short incision or MIS with tool-kit, using computer-generated block randomization (block of four). After randomization, sequentially numbered, opaque, sealed envelopes prepared by a person not involved in the trial were used in the allocation concealment process. The envelopes were opened just prior to skin preparation by the surgeon. All surgical procedures were performed by an experienced hand surgeon (PA) who was not involved in the outcome assessment process, and all patients were advised not to observe the operation. Thus, patients, allocator and outcome assessor were blinded to the study.

### Preoperative preparation

The operations were performed in a minor operation room. All patients received 5–10 mL of 1% lidocaine with adrenaline 1:200,000 subcutaneous injection as wide-awake local anesthesia with no tourniquet (WALANT).

### *Short incision group* [18]

**Fig. 1:**
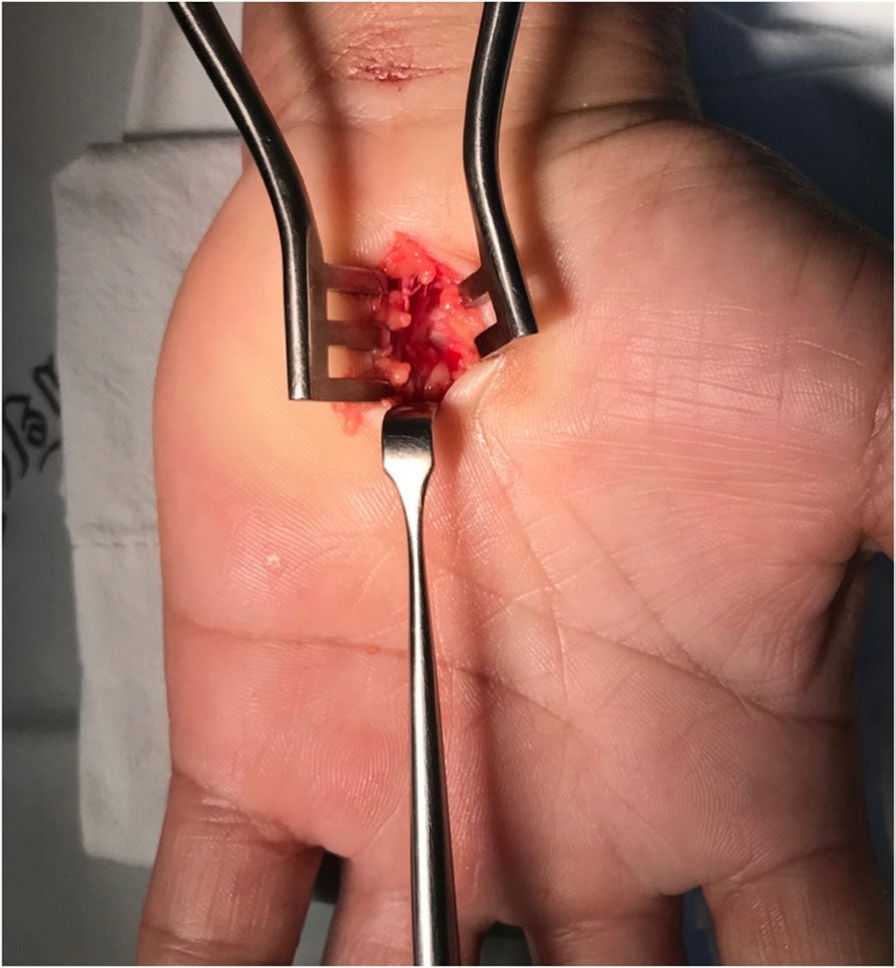
Short incision showing complete transverse carpal ligament release under direct observation

A 2.5 to 4.0-cm-long incision was made along the line of the radial axis of the ring finger from Kaplan’s cardinal line to the area distal to the transverse wrist crease. After that, the subcutaneous tissue was bluntly dissected with a Stevens scissor and retractors were used to separate the borders of the incision. The palmar fascia was identified and divided. Then the transverse carpal ligament was identified and the mid portion of the ligament was incised with a no. 15 scalpel to provide access to the carpal tunnel. Stevens scissors were used to sever the distal and proximal portion of the transverse carpal ligament. Complete transverse carpal ligament release was confirmed by direct observation using a Ragnell retractor to maintain a clear surgical field (Fig. 1). The incisions were closed with vertical mattress 4/0 nylon sutures and a pressure bandage was applied.

### *MIS with tool-kit group* [19]

**Fig. 2:**
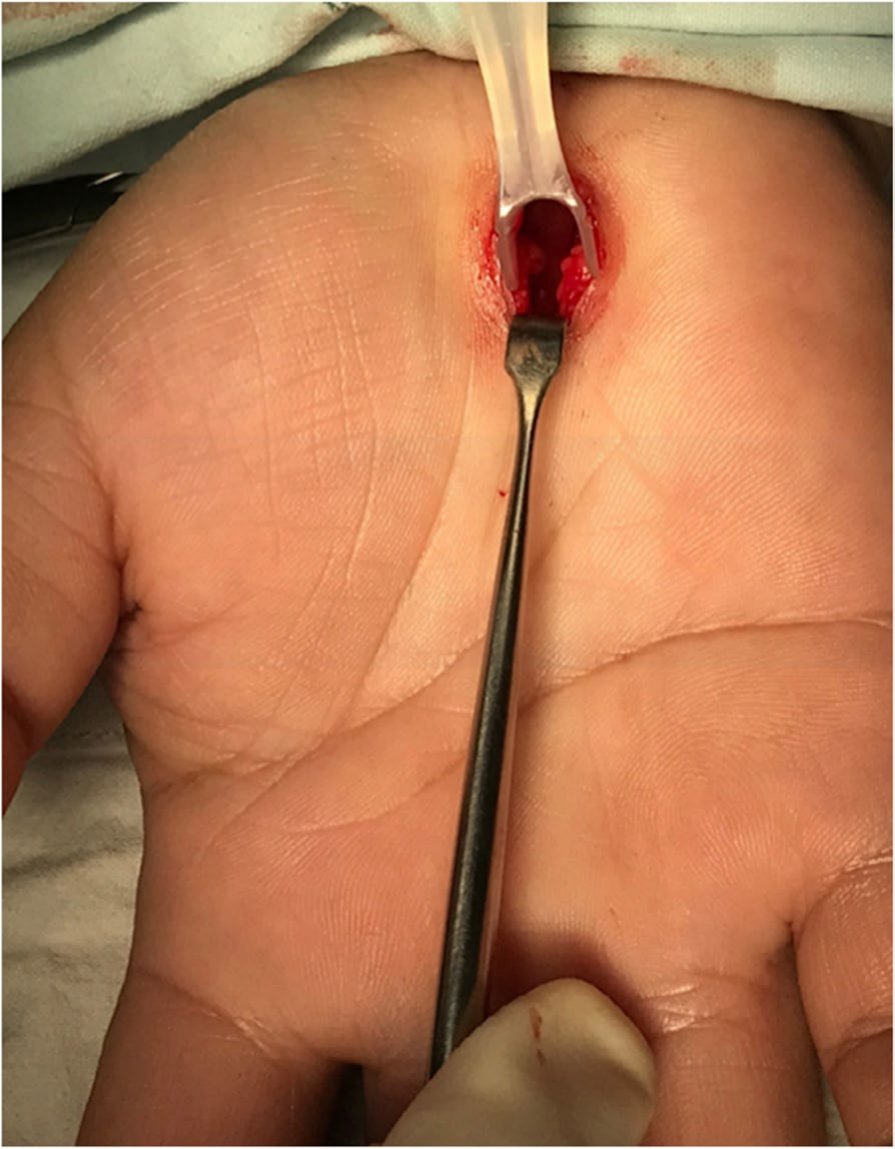
MIS with tool-kit technique showing complete transverse carpal ligament release under direct observation

We used the third generation of MIS-CTS kits (ProMIS®) which was developed by Wongsiri et al. from Prince of Songkla University, Hat Yai, Tailand for MIS carpal tunnel release with Wongsiri technique [19]. A 1.5–2.0 cm incision was made 2.0 cm distal to the wrist crease along the line of the radial axis of the ring finger. Subcutaneous tissue and the palmar aponeurosis were then bluntly dissected using a Stevens scissor. The navigator tip of a MIS-CTS View was inserted to create a working space just above the retinaculum area located on top of the transverse carpal ligament. The visual tube of the MIS-CTS View was inserted along the working space that had been created. Therefore, the transverse carpal ligament could be viewed obviously under the visual tube. The distal portion of the transverse carpal ligament was incised with a no.15 scalpel to enter the carpal tunnel. The freer of the MIS-CTS Cut was inserted into the carpal tunnel to free the median nerve from adhesion to the transverse carpal ligament. The knife of the MIS-CTS Cut was applied to completely and smoothly separate the transverse carpal ligament from the distal to the proximal portion. Complete transverse carpal ligament release was confirmed under direct observation via the visual tube of the MIS-CTS (Fig. 2). Skin closure was performed as described above.

### Postoperative protocol

Patients were allowed to move their wrists, hands and fingers the same day that the surgery was performed. No wrist splint was needed. Non-steroidal anti-inflammatory drugs or tramadol (oral form) were taken for a few days for postoperative pain control. Oral antibiotics were administered for a week. The original dressing and the surgical stitches were removed after 12 days.

### Standard rescue protocol

In cases where complete transverse carpal ligament release could not be confirmed due to limited direct visualization, the incision was extended proximally to the wrist crease (standard incision).

### Outcome measurement

The primary outcomes were postoperative anterior wrist pain at the 2nd week (postoperative days 12–14) and the time to return to work or activities of daily living. Postoperative anterior wrist pain was defined as pain from the surgical wound, scar or pillar pain [30] that caused the patient discomfort. We evaluated anterior wrist pain using the visual analogue scale (VAS) both at rest and while conducting daily activities. We used the VAS instrument which had 11-point Numerical Rating Scale [31]. Time to return to work and to activities of daily living were measured from postoperative day 1 to the day the patient could perform at the same level as the day before surgery.

Secondary outcomes were functional and symptom severity improvement which were evaluated using the Thai Michigan hand questionnaire at the day before surgery and at the 2nd week postoperatively (postoperative days 12–14) [32].

### Statistical analysis

The sample size was estimated using the formula for a randomized controlled trial for continuous data according to the study of Saw et al. [33]. The average VAS scale intensity of anterior wrist pain in the short incision group at the 2nd week postoperatively was 2.5 with standard deviation (SD) = 0.6. The minimal clinically significant difference of musculoskeletal pain intensity was 1 [34]. We hypothesized that MIS with tool-kit results in clinically significantly lower postoperative anterior wrist pain (VAS = 1.5) than short incision.

Estimated sample sizes for a two-sample means test.

t test assuming SD1 = SD2 = SD.

Ho: m2 = m1 versus Ha: m2! = m1.

Study parameters: alpha = 0.05, power = 0.80, delta = − 1.00, m = 2.5, m2 = 1.5, SD = 0.6.

Estimated sample sizes: total number = 14, number per group = 7.

A total of 7 patients in each group were needed to achieve 80% statistical power at the 5% significance level to detect the size of the input effect. To compensate for potential patient dropouts, we used a sample size of 12 patients per group. There is no generally accepted minimal clinically significant difference in time to return to work or activities of daily living. For that reason, we used the sample size mentioned above. We hypothesized that the time to return to work or to activities of daily living would be correlated with postoperative anterior wrist pain, i.e., the less postoperative anterior wrist pain, the earlier the return to work and/or activities of daily living.

The trial compared the outcomes in patients who underwent MIS with tool-kit and those who had a short incision in terms of postoperative anterior wrist pain, time to return to work or activities of daily living, and functional and symptom severity improvement. Continuous variables, i.e., postoperative anterior wrist pain, time to return to activities of daily living and functional and symptom severity improvement, were evaluated using the Mann-Whitney rank-sum test as the data were nonnormally distributed. Continuous variables, including time to return to work and wound length, were assessed using the Student’s t-test as the data were normally distributed. Statistical analysis was performed using Stata Statistical software 15 (Stata Corp, LP, College Station, TX). A *P*-value of less than 0.05 was considered statistically significant.

## Results

### Population

**Fig. 3:**
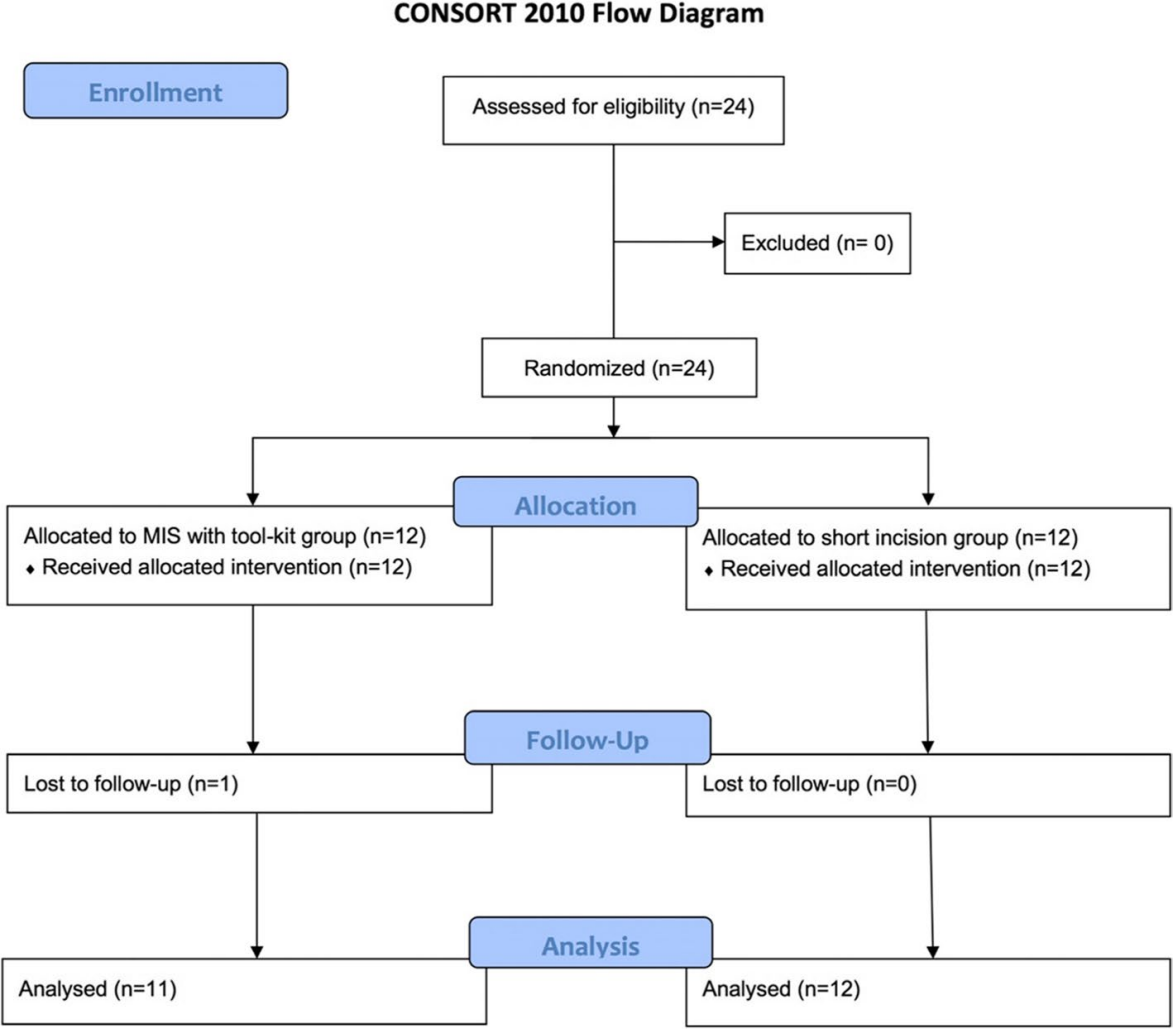
CONSORT flow diagram

All enrolled patients (*n* = 24) were assigned to one of two groups, either the short incision group or the MIS with tool-kit group in a 1:1 ratio. After randomization, the number of patients in both groups was equal (12 patients per group). One patient in the MIS with tool-kit group failed to appear at the scheduled follow up and was dropped from the analysis (Fig. 3).

There were no significant differences in demographic data between the MIS with tool-kit and the short incision groups in terms of age (*P* = 0.94), sex (*P* = 0.22), dominant hand (*P* > 0.99), affected hand (*P* = 0.68), severity as determined by electrodiagnosis measurement (P > 0.99), underlying diabetes mellitus (*P* = 0.48) or occupation (*P* = 0.49) (Table 1).

**Table 1:** Patient characteristics of the MIS with tool-kit and short incision groups before surgery

Characteristics	All patients	MIS with tool-kit	Short incision	***P***-value
	(***n*** = 23)	(***n*** = 11)	(***n*** = 12)	
Age, mean (SD), years	54 (14)	53 (9)	54 (17)	0.94
Sex, n (%)				0.22
Women	20 (92)	11 (100)	9 (75)	
Men	3 (8)	0 (0)	3 (25)	
Dominant hand, n (%)				> 0.99
Right	20 (87)	10 (91)	10 (83)	
Left	3 (13)	1 (9)	2 (17)	
Affected hand, n (%)				0.68
Right	11 (48)	6 (55)	5 (42)	
Left	12 (52)	5 (45)	7 (58)	
Severity, n (%)				> 0.99
Mild	0 (0)	0 (0)	0 (0)	
Moderate	4 (33)	2 (18)	2 (17)	
Severe	19 (67)	9 (82)	10 (83)	
Underlying Diabetes Mellitus, n (%)				0.48
Yes	2 (9)	0 (0)	2 (17)	
No	21 (91)	11 (100)	10 (83)	
Occupation, n (%)				0.49
Manual laborer	8 (35)	3 (27)	5 (41)	
Officer	4 (17)	2 (18)	2 (17)	
Housewife	7 (30)	5 (46)	2 (17)	
Unemployed	2 (9)	0 (0)	2 (17)	
Retired	2 (9)	1 (9)	1 (8)	

*SD* Standard deviation

### Clinical outcomes

There was no significant difference between the MIS with tool-kit and the short incision groups in the primary outcomes: VAS of anterior wrist at rest (0(0–0) vs 0(0–0), P > 0.99) and while conducting daily activities (0(0–1) vs 0(0–1), *P* = 0.89) and time to return to activities of daily living (7 (SD 4) days vs 6 (SD 3) days, *P*=0.46) and work (14 (SD 8) days vs 11 (SD 5) days, *P*=0.24). MHQ score improvement, the secondary outcome, showed no significant difference between the groups (9 (2-31) vs 13 (1-32), *P*=0.95). The MIS with tool-kit group, however, did have a significantly shorter wound length than the short incision group (1.95 (SD 0.47) cm vs 2.92 (SD 0.56) cm, *P* < 0.01) (Table 2). No adverse events were observed. The results of each patient were shown in Supplement 1.

**Table 2:** Comparison of results after carpal tunnel release in the MIS with tool-kit and the incision groups

Group	MIS with tool-kit	Short incision	*P*-value
	(*n* = 11)	(*n* = 12)	
VAS on anterior wrist at 2 weeks after surgery, median (IQR)			
Rest	0 (0–0)	0 (0–0)	> 0.99
Doing daily activity	0 (0–1)	0 (0–1)	0.89
MHQ score improvement, median (IQR)	9 (2–31)	13 (1–32)	0.95
Wound length, mean (SD), cm	1.95 (0.47)	2.92 (0.56)	< 0.01
Return to daily activity, mean (SD), day	7 (4)	6 (3)	0.46
Return to work, mean (SD), day	14 (8)	11 (5)	0.24

*SD* Standard deviation

## Discussion

This randomized controlled trial demonstrated that there were no differences in terms of early postoperative anterior wrist pain or time to return to work or to activities of daily living between the MIS with tool-kit and the short incision patients who underwent CTS release. Our results differ significantly from a recent meta-analysis [15]. Among the reasons for the difference might be the relatively small difference in wound length between the MIS with tool-kit and the short incision groups (1.95 (SD 0.47) cm vs 2.92 (SD 0.56) cm) in this study which had small effect size. Most studies included in previous meta-analyses compared standard long curvilinear palmar incisions proximal to the distal wrist crease had wound lengths up to 5–7 cm. vs. the MIS techniques which had significant difference in surgical incision length. The much smaller wound lengths with the MIS technique compared to those with the standard technique could result in less wound and/or scar pain leading to a shorter time to return to normal activities in the MIS group [15, 18, 24–26]. Differences in ethnicity and culture could also potentially affect pain sensitivity, pain perception and pain threshold in different populations [35–43], therefore the anterior wrist pain at 2nd week after operation in our trial was quite low compared to the previous publications [18, 33]. Studies evaluating pain sensitivity, perception and thresholds in the Thai population and in populations of other ethnicities are still needed.

The result in term of time to return to work in our study was similar to the randomized controlled trials which compared MIS with double small incision technique (1-cm transverse proximal incision+ 1.5-cm distal incision) to endoscopic technique (double 1.0-cm incisions) for CTS release [24]. The time return to work in MIS with double small incision and endoscopic groups were 14 (SD 8) and 12 (SD 9) days, respectively without the statistical significance (*P* = 0.165). We realized that comparable results occurred because of both MIS with double small incision and endoscopic groups had the small difference of surgical incision length (small effect size) similar to our study. The different social security systems, with potential entitlement to paid sick leave in different countries might affect the time to return to work [44].

In this trial, no patients had their dressing changed prior to the 2nd week postoperatively and postoperative anterior wrist pain was evaluated prior to removal of the dressing to ensure that all patients remained blinded regarding the type of operation performed. No patients took the oral pain-control medication for more than 3 days after the operation, so the effect of oral pain-control medication did not affect the evaluation of postoperative anterior wrist pain at the 2nd week after surgical release. As both surgical techniques provided adequate visualization, no extended incision proximal to the wrist crease was performed with any of the patients.

The short incision technique provided satisfactory outcomes in terms of anterior wrist pain and time to return to daily activities and work and were comparable to the MIS with tool-kit technique. The short incision technique did not require any special instruments which offered the benefit in term of cost-effectiveness unlike the MIS technique which required special tools to perform the complete transverse carpal ligament release under direct visualization. However, the MIS with tool-kit technique had the advantage of creating a smaller wound length and thus a smaller scar, making it preferable for patients concerned about cosmetic appearance and who could afford the extra cost.

There are several limitations in this randomized controlled trial. First, the study only compared selected aspects between the MIS with tool-kit and the short incision approaches. Other aspects, e.g., satisfactory, grip or pinch strength, may have yielded different results. Second, the sample size was relatively small. Although the sample size of our study was adequate to interpret the primary outcomes, a prospective randomized controlled trial involving a larger population and a long-term follow-up is needed to confirm our results. Third, there is a limitation in terms of generalizability as this study included only Thais. Additional studies should be conducted in other populations. Fourth, after removal of the dressing, patients might have been able to discern the type of surgical technique they had received which could have biased the results for time to return to work and to activities of daily living. Fifth, since we used block of four in the method of randomization, the small block size could increase the risk that the allocation process might be predictable.

## Conclusions

There are no differences in terms of early postoperative anterior wrist pain or in time to return to work or activities of daily living between the short incision and the MIS with tool-kit techniques in patients undergoing CTS release. Short incision is recommended for benefit in term of cost-effectiveness, while the MIS with tool-kit method results in less surgical wound length which could be important for patients concerned with cosmetic appearance.

## Supplementary Information

**Additional file 1.** Results after carpal tunnel surgery in each group.

## Data Availability

The datasets used and/or analysed during the current study are available from the corresponding author on reasonable request.

## Abbreviations

*cm*: *Centimeter*
CONSORT: Consolidated Standards of Reporting Trials
CTS: Carpal tunnel syndrome
MHQ: Michigan hand questionnaire
MIS: minimally invasive surgery
SD: Standard deviation
VAS: Visual analogue scale
WALANT: wide-awake local anesthesia with no tourniquet
