# Supplementary material for: Short incision versus minimally invasive surgery with tool-kit for carpal tunnel syndrome release: a prospective randomized control trial to evaluate the anterior wrist pain and time to return to work or activities
Source: BMC Musculoskelet Disord. 2022 Jul 25;23:708. doi: 10.1186/s12891-022-05663-5 (PMC9316708; doi:10.1186/s12891-022-05663-5)
Supplement: Supplementary file 1 — Additional file 1. Results after carpal tunnel surgery in each group. [file 12891_2022_5663_MOESM1_ESM.docx]

**Supplement 1** Results after carpal tunnel surgery in each group

MIS with tool-kit group

| case | VAS on anterior wrist at 2 weeks after surgery | | MHQ score improvement | Wound length, cm | Return to daily activity, day | Return to work, day |
| --- | --- | --- | --- | --- | --- | --- |
|  | Rest | Doing daily activity |  |  |  |  |
| 1 | 0 | 0 | 3.9 | 2.0 | 3 | 12 |
| 2 | 0 | 0 | 37.7 | 2.0 | 5 | 7 |
| 3 | 4 | 4 | 36.0 | 2.0 | 7 | 8 |
| 4 | 0 | 5 | 2.3 | 2.0 | 7 | 14 |
| 5 | 0 | 0 | 30.7 | 1.5 | 14 | 30 |
| 6 | 0 | 0 | 27.9 | 1.0 | 14 | 21 |
| 7 | 0 | 0 | 2.3 | 2.0 | 2 | 4 |
| 8 | 0 | 1 | -3.1 | 2.0 | 7 | 14 |
| 9 | 0 | 1 | 9.0 | 2.0 | 7 | 15 |
| 10 | 0 | 0 | 0.4 | 3.0 | 7 | 20 |
| 11 | 0 | 0 | 29.6 | 2.0 | 2 | 7 |

Short incision group

| case | VAS on anterior wrist at 2 weeks after surgery | | MHQ score improvement | Wound length, cm | Return to daily activity, day | Return to work, day |
| --- | --- | --- | --- | --- | --- | --- |
|  | Rest | Doing daily activity |  |  |  |  |
| 1 | 5 | 5 | 27.7 | 4 | 5 | 14 |
| 2 | 0 | 2 | 3.8 | 3 | 10 | 20 |
| 3 | 0 | 0 | 34.8 | 3 | 3 | 7 |
| 4 | 0 | 0 | -1.0 | 3.5 | 5 | 10 |
| 5 | 0 | 1 | 43.5 | 3 | 2 | 5 |
| 6 | 0 | 0 | 37.0 | 3 | 7 | 14 |
| 7 | 0 | 0 | -10.2 | 2 | 8 | 10 |
| 8 | 0 | 0 | 14.2 | 3 | 10 | 15 |
| 9 | 0 | 0 | 29.1 | 3 | 4 | 5 |
| 10 | 0 | 0 | 9.9 | 3 | 5 | 7 |
| 11 | 0 | 1 | -0.8 | 2.5 | 5 | 7 |
| 12 | 0 | 1 | 10.9 | 2 | 5 | 14 |
